# Persistence of Uterine Artery Doppler Velocimetry Changes in Postpartum Mothers Following a Delivery Complicated by Preeclampsia: A Systematic Review

**DOI:** 10.7759/cureus.74497

**Published:** 2024-11-26

**Authors:** Prathamesh Lanjewar, Sivaranjani P Selvam, Avir Sarkar, Shivam Pandey

**Affiliations:** 1 Obstetrics and Gynaecology, Noida International Institute of Medical Sciences, Noida, IND; 2 Obstetrics and Gynaecology, Jawaharlal Institute of Postgraduate Medical Education and Research, Puducherry, IND; 3 Department of Biostatistics, All India Institute of Medical Sciences, New Delhi, IND

**Keywords:** diastolic notch, postpartum period, preeclampsia, uterine artery doppler, uterine artery pulsatility index

## Abstract

In preeclampsia, there occurs a defective trophoblastic invasion of spiral arteries, which is characterized by abnormal uterine artery wave parameter such as increased pulsatility index (PI) and early diastolic notch. This increased uterine artery PI is a good predictor of hypertensive disorder and small for gestational-age babies. Maternal hypertension and proteinuria resolve in the puerperium. Arterial Doppler changes in normal pregnancy were extensively studied. Doppler changes in uterine artery in hypertensive postpartum women were less researched and unclear. This review aimed to compare the uterine artery Doppler changes in preeclamptic and normotensive women during the puerperium through an extensive search of the available literature. It was found that even with the lengthiest follow-up of six weeks postpartum, the increased uterine artery PI persisted and the magnitude was higher in hypertensive than normotensive postpartum women.

## Introduction and background

In normal pregnancy, the spiral arteries get invaded by endovascular trophoblasts, resulting in enormous changes in the uterine vasculature. With these changes, the high resistance, low flow pattern changes to low resistance, high flow pattern, thus establishing a good maternal-fetal circulation. But in the case of preeclampsia, there occurs defective trophoblastic invasion of spiral arteries, which is characterized by abnormal uterine artery wave parameter such as increased pulsatility index (PI) and early diastolic notch [1]. Preeclampsia is defined as an increase in blood pressure of more than 140/90 mmHg on two occasions four hours apart at a gestation greater than 20 weeks and is detected for the first time in pregnancy. Blood pressure generally normalizes by six weeks postpartum. This increased uterine artery PI is a good predictor of hypertensive disorder and small for gestational-age babies [2]. Maternal hypertension and proteinuria resolve in the puerperium. Uterine involution involves changes in the uterine artery Doppler in addition to the changes in the musculature and endometrium. Arterial Doppler changes in normal pregnancy were extensively studied. Many studies found an increase in uterine artery PI in the puerperium [3] whereas few studies did not find any significant difference [4]. Doppler changes in uterine artery in hypertensive postpartum women are less researched and unclear. This review aimed to compare the uterine artery Doppler changes in preeclamptic and normotensive women during the puerperium through an extensive search of the available literature. It analyzes whether uterine artery Doppler velocimetry changes noticed during preeclampsia persist even in the postpartum period. Data from the various original articles that were pooled and included in this review showed that the increased uterine artery PI persisted in the postpartum period beyond six weeks and the magnitude was higher in the hypertensive women than the normotensive group.

Thus patients with preeclampsia are prone to developing chronic hypertension if they are not treated in the postpartum period also. In a practical scenario, uterine artery PI measurements at six weeks postpartum can help in triaging the women and identifying those who are prone to developing chronic hypertension and treating them with additional antihypertensives accordingly. 

## Review

In this review, we aimed to assess whether uterine artery Doppler velocimetry changes in women with preeclampsia persists in the postpartum period. PROSPERO (an international database of prospectively registered systematic reviews in health and social care) registration was done well before conducting the systematic review (CRD42022345548) [5]. The Preferred Reporting Items for Systematic Reviews and Meta-Analyses (PRISMA) 2020 flowchart was followed for conducting this review [6]. A detailed literature search was conducted and all data pertaining to uterine artery Doppler velocimetry in postpartum women was retrieved.

*Research question:* Did uterine artery Doppler changes continue to persist in the postpartum period in mothers following pregnancy complicated with preeclampsia? 

**Figure 1:**
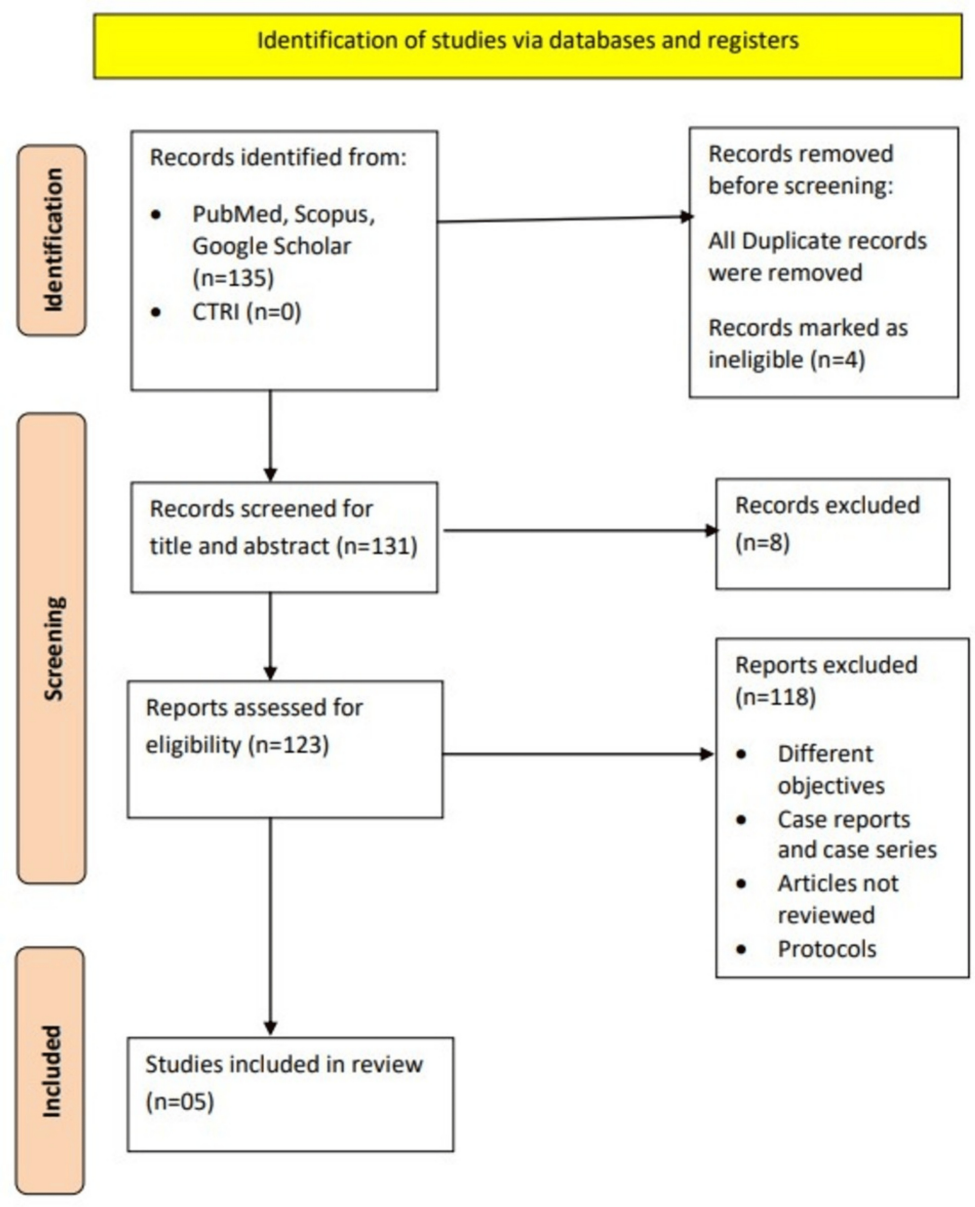
PRISMA flowchart used in this systematic review PRISMA: Preferred Reporting Items for Systematic Reviews and Meta-Analyses; CTRI: Clinical Trials Registry of India

*Systematic search: *A pre-defined search with the following keywords: ((Uterine artery Doppler) AND (postpartum)) was used to search articles published in PubMed, Scopus, Google Scholar and Clinical Trial Registries till July 2023. Literature search found 135 studies. The database search was carried out as follows: (a) Uterine artery Doppler (2,873); (b) postpartum (126,487); #a OR #b (n=135). After retrieving the abstracts, 131 studies were included. All non-relevant studies were excluded and 123 studies were found to be eligible for assessment. The final analysis led to the acceptance of five articles in this systematic review (Figure 1).

*Inclusion criteria:* (1) Studies assessing the Doppler velocimetry changes in the uterine artery in postpartum mothers after a pregnancy complicated with preeclampsia and (2) randomized controlled trials; (3) retrospective and prospective cohort studies, and (4) case-control studies. 

*Exclusion criteria:* (a) Case reports and case series articles, (b) editorial articles, (c) articles in pre-print, (d) non-English articles, (e) studies assessing uterine artery Doppler only in postpartum women after an uncomplicated pregnancy (not including preeclamptic mothers).

Two authors (PL and SPS) did an independent search of all the relevant articles. Similarity indices were checked. Any disagreement between the decisions of the two authors regarding the inclusion or exclusion of text was settled after obtaining consensus from the third and fourth authors (AS and SP). Information was collected on the objective of the study, its design, sample size, variables related to age, parity, body mass index (BMI), mean blood pressures, antenatal and postpartum uterine artery notching and PI. 

Results

Out of the included studies, four were prospective cohort studies and one was a case-control study. One was from Israel [7], one from Korea [8], one from Egypt [9], one from Portugal [10], and the other from Toronto, Canada [11]. The sample size ranged from 32 to 200. In all the studies, uterine artery PI was measured and the presence of an early diastolic notch was also noted. In all the studies postpartum uterine artery PI measurement and the presence of early diastolic notch was done by transvaginal ultrasonography whereas Weintraub et al. used transabdominal sonography [7]. The study by Lee et al. was a cross-sectional study where uterine artery PI was measured once in the antepartum period and again in the immediate or late postpartum period [8]. All other studies were longitudinal prospective studies. All the studies in this review compared normotensive women with those having preeclampsia. Guedes-Martin et al. compared normotensive and chronic hypertensive women, excluding those with preeclampsia [10]. Lee et al. included chronic hypertensives along with those with preeclampsia [8]. Weintraub et al. measured uterine artery PI only in the postpartum period. Antepartum uterine artery PI was taken retrospectively from the records [7]. The other studies measured uterine artery PI both in the antepartum and postpartum period though the timing differed in all the studies. The postpartum measurement by Weintraub et al. [7] and Maged et al. [9] were in the immediate postpartum period, that is, 51 hours and 24-72 hours post-delivery, respectively. The maximum post-partum timing was six weeks by Naeh et al. [11]. In all the studies, it was observed that the uterine artery PI was higher in the hypertensive group both in the antepartum and postpartum periods. The presence of early diastolic notch was observed more among the hypertensive group of women in the postpartum period. Maged et al. compared the difference in uterine artery PI of antepartum and postpartum women in both the groups and found that there was no difference between the two [9]. Guedes-Martin et al. observed an increase in uterine artery PI in both the groups, but the magnitude was higher in the hypertensive groups [10]. They also highlighted that genetic basis may influence uterine artery perfusion irrespective of the pathological conditions. The summary and key findings of the studies are tabulated in Table 1.

**Table 1:** Summary of the results of the studies included in the review PI: Pulsatility index; RI: resistivity index; FGR: fetal growth restriction

S. no.	Study	Study Design	Country	Sample size	Parameters studied	Key findings	Inference
1.	Weintraub et al. (2013) [7]	Prospective cohort study	Israel	Severe preeclampsia (n=31) and normotensive controls (n=52)	Comparison of Uterine artery PI among preeclamptic vs normotensive women in the postpartum period	Uterine artery PI was measured 51.2 hours after delivery. Comparison was made between preeclamptic and normotensive women.	The presence of unilateral and bilateral early diastolic notches in the postpartum period were significantly higher in patients with severe preeclampsia
2.	Lee et al. (2016) [8]	Cross- sectional study	Korea	122 women; Group 1 (women with hypertension): 11 antenatal, 13 immediate postpartum and 10 late postpartum; Group 2 (normotensive women): 32 antenatal, 29 immediate postpartum and 27 late postpartum	Comparison of uterine artery PI among (preeclamptic + eclamptic + chronic hypertensive) vs normotensive women in both antenatal and postpartum period	Uterine artery PI was measured in antenatal period (24-40 weeks), immediate and late postpartum (three to five weeks)	Uterine artery PI in hypertensive pregnancies was higher than in normal pregnancies in puerperium, suggesting that it takes several weeks for hypertensive vascular changes to normalize (more than five weeks) after delivery
3.	Maged et al. (2019) [9]	Prospective cohort study	Egypt	100 women with preeclampsia and 100 normotensive controls	Comparison of uterine artery PI and resistive index (RI) among preeclamptic vs normotensive women in both antenatal and postpartum period	Comparison of uterine artery PI and RI was done between preeclamptic vs normotensive women in both antenatal and postpartum periods	Uterine artery PI and RI were significantly higher in women with preeclampsia than normotensive women, both in antenatal and postpartum period
4.	Guedes-Martins et al. (2015) [10]	Prospective cohort study	Portugal	24 hypertensive women (cases) and 28 normotensive women (controls)	Comparison of uterine artery PI among chronic hypertensive vs normotensive women in both antenatal and postpartum periods	Uterine artery PI was measured just before the caesarean section, at one week and four weeks postpartum	Chronic hypertensive women with normal pregnancy outcomes exhibited a progressively increasing postpartum uterine artery impedance. This trend is higher in hypertensive women than in normotensive women.
5.	Naeh et al. (2022) [11]	Prospective cohort study	Toronto, Ontario, Canada	15 women with preeclampsia or fetal growth restriction (FGR) (cases) and 17 normotensive women (controls)	Comparison of uterine artery PI among preeclamptic vs normotensive women in both antenatal and postpartum period	Uterine artery PI was measured at 18-22 weeks gestation and six weeks postpartum	Women with preeclampsia or FGR had significantly higher uterine artery PI in both antenatal and postpartum period

*Methodological quality of the studies:* A quality check of all the included studies was done using Joana Brigg's Institute (JBI) critical appraisal checklist. The risk of bias was assessed for the four cohort studies and is tabulated in Table 2. Since the fifth study (Lee et al.) is a cross-sectional study, the JBI checklist could not be applied in this given formal for that study [8]. All studies were found to be at a low risk for bias. 

**Table 2:** Quality assessment of included cohort studies using Joana Brigg's Institute (JBI) critical appraisal checklist

Quality Check Parameters	Weintraub et al. [7]	Maged et al. [9]	Guedes-Martins et al. [10]	Naeh et al. [11]
1. Were the two groups similar and recruited from the same population?	Yes	Yes	Yes	Yes
2. Were the exposures measured similarly to assign people to both exposed and unexposed groups?	Yes	Yes	Yes	Yes
3. Was the exposure measured in a valid and reliable way?	Yes	Unclear	Yes	Yes
4. Were confounding factors identified?	Unclear	Unclear	Unclear	Unclear
5. Were strategies to deal with confounding factors stated?	Unclear	Unclear	Unclear	Unclear
6. Were the groups free of the outcomes at the start of the study?	Yes	Yes	Yes	Yes
7. Were the outcomes measured in a valid and reliable way?	Yes	Yes	Yes	Yes
8. Was the follow up time reported and sufficient to be long enough for outcome to occur?	No	Yes	Yes	Yes
9. Was follow-up complete?	Yes	Yes	Yes	Yes
10. Were strategies to address incomplete follow-up utilized?	Unclear	Unclear	Unclear	Unclear
11. Was appropriate statistical analysis used?	Yes	Yes	Yes	Yes

Discussion

The alterations in the uterine vasculature can be assessed with Doppler ultrasonography [12-14]. The uterine vasculature undergoes vigorous changes in different stages in the reproductive phase of a woman, and as in the non-pregnant state, the changes are due to the variation in estrogen and progesterone levels. During pregnancy, the remarkable change is the disappearance of an early diastolic notch and a continuous diastolic flow [14]. High resistance and low flow vasculatures get converted into low resistance, high flow vascular channels due to trophoblastic invasion of spiral arteries. Defective trophoblastic invasion of spiral arteries, as is evident in hypertensive women, is depicted as early diastolic notch and increased uterine artery PI in the uterine artery Doppler wave pattern. Soon after delivery, low resistance and high capacitance vasculature is no longer required and the luminal obliteration occurs with thrombosis, arteritis and thickening of the intima [15]. This luminal obliteration leads to an increase in the vascular resistance depicted as increased uterine artery PI even in normotensive women as observed by Guedes-Martin et al. [10,16]. So, even in normotensive women, postpartum uterine artery PI was found to be higher, implying that these changes occurred to attain the pre-pregnant state. The same study stated that the magnitude of the increase in uterine artery PI was higher in the hypertensive group. Maternal hypertension and proteinuria settled in the puerperium, but whether the uterine artery PI changes resolved in the puerperium was unclear. All the five studies observed an increase in the uterine artery PI in the puerperium. Guedes-Martin et al. also observed that there was an increase in the uterine artery PI in both normotensive and hypertensive women, but the magnitude was higher among hypertensives [10]. Even the study involving the late postpartum period by Naeh et al. observed an increase in uterine artery PI at six weeks postpartum [11].

The reasons stated for the non-resolution of uterine artery PI in the puerperium are: (a) the defective trophoblastic invasion of the decidua basalis and myometrium which persist even after the placental delivery [12,17]; (b) atherosclerosis of the spiral arteries in the placental bed to which placental insufficiency can be ascribed to [13]; and (c) the constantly increased vascular tone in hypertensives, which may influence the uterine artery impedance. The maximum period of postpartum follow-up among these five studies was six weeks where the increased uterine artery PI persisted. So further studies involving the long-term follow-up is required for a better understanding of the postpartum uterine artery Doppler waveforms. This may lead to further research about the association of increased postpartum uterine artery PI and the long-term cardiovascular and neurological consequences.

This systematic review had few strengths. All five studies were prospective. Data collection and analysis were meticulously done. In almost all the studies, the sonographic examination was done by the same examiner, eliminating the inter-observer bias. Maged et al. had an adequate sample size of 200 and they followed the same patient in the antepartum and postpartum period longitudinally [9]. In that study, all women delivered vaginally, eliminating the difficulty in mobility and ultrasound examination difficulties encountered in caesarean deliveries. Guedes-Martin et al. included only those underwent elective caesarean deliveries eliminating the confounding factors related to mode of delivery, labor-related variables like dystocia and first stage of labor [10]. Maged et al. excluded caesarean deliveries reasoning that spinal anesthesia decreases the uterine artery PI in hypertensives [9]. This systematic review was the first of its kind (to our knowledge), which may lead to many studies involving the association of postpartum uterine artery Doppler changes and the long-term cardiovascular and neurological consequences.

The study had a few limitations. All studies were single-centered. The sample size was least in the study by Naeh et al. [11]. The uterine artery PI measurement was in the immediate postpartum period by Weintraub et al., whereas actually the changes persist several weeks postpartum.

## Conclusions

Deranged uterine artery Doppler velocimetry was more pronounced in hypertensive women compared to normotensive controls both in antenatal and postpartum period. Even with the lengthiest follow-up among these studies, that is, six weeks postpartum, the increased uterine artery PI persisted and the magnitude was higher in the hypertensive women than in the normotensive group. Thus patients with preeclampsia are prone to developing chronic hypertension if they are not treated in the postpartum period also. In a practical scenario, uterine artery PI measurements at six weeks postpartum can help in triaging the women and identifying those who are prone to developing chronic hypertension and treating them with additional antihypertensives accordingly. Further studies involving the long-term follow-up is required for better understanding of the postpartum uterine artery Doppler waveforms.
